# Dietary fiber influence on overall health, with an emphasis on CVD, diabetes, obesity, colon cancer, and inflammation

**DOI:** 10.3389/fnut.2024.1510564

**Published:** 2024-12-13

**Authors:** Layla A. Alahmari

**Affiliations:** Department of Community Health, College of Applied Medical Sciences, Northern Border University, Arar, Saudi Arabia

**Keywords:** dietary fiber, obesity, cardiovascular disease, diabetes, colon cancer

## Abstract

Dietary fiber, found in plant-based foods, plays an essential role in human health. It is divided into two types—soluble and insoluble—both offering significant health benefits. Research has shown that increasing fiber intake can reduce the risk of various chronic diseases, such as cardiovascular diseases (CVD), type II diabetes, obesity, colon cancer, and inflammation. These health conditions are major global challenges, making fiber consumption a key focus for disease prevention. This study reviews a range of clinical trials, cohort studies, and meta-analyses to explore how dietary fiber affects these health risks. By synthesizing data from multiple sources, we found a clear association between higher fiber intake and a lower incidence of these diseases. However, studying the effects of fiber on health presents several challenges. Variations in fiber types and bioavailability make it difficult to generalize results. Additionally, dietary intake is often self-reported, leading to potential inaccuracies in data. Many studies also lack consistency in methodology, and short study durations limit the ability to assess long-term health outcomes. These factors make it harder to draw definitive conclusions about the full range of fiber’s health benefits. Despite these challenges, increasing fiber-rich foods like fruits, vegetables, whole grains, and legumes remains a highly recommended strategy for improving health and reducing the risk of chronic disease.

## 1 Introduction

Dietary components such as fats, carbohydrates, proteins, vitamins, minerals, and fibers are essential for maintaining health, with their consumption varying based on factors like age, gender, physical activity, and individual health needs (1). Among these, dietary fibers play a crucial role in supporting various physiological functions. Fibers are primarily classified into two categories: soluble fibers (e.g., pectin, gums) and insoluble fibers (e.g., cellulose, lignin), each of which has distinct physiological effects. While soluble fibers are known for their role in regulating blood sugar levels and promoting gut health, insoluble fibers contribute to digestive health by enhancing bowel regularity and may reduce the risk of certain cancers (2, 3).

The primary sources of dietary fibers include fruits, vegetables, whole grains, and legumes, which provide a range of fiber types with diverse health benefits. Despite their well-established roles in supporting digestive health and preventing constipation, the potential of dietary fibers to prevent or manage chronic diseases such as cardiovascular diseases (CVD), type II diabetes, obesity, and colon cancer has garnered significant research interest (4–6). However, while many studies highlight the broad health benefits of dietary fibers, the exact mechanisms through which they influence these conditions remain poorly understood, presenting a significant gap in the existing literature.

Cardiovascular diseases (CVD), which include conditions such as heart disease, stroke, and hypertension, are among the leading causes of morbidity and mortality worldwide (7). Emerging evidence suggests that dietary fibers, particularly soluble fibers, may help reduce cholesterol levels, improve lipid profiles, and enhance endothelial function, thereby reducing the risk of CVD. Type II diabetes, characterized by insulin resistance and chronic hyperglycemia, is another condition influenced by dietary fiber intake (8, 9).

Studies have shown that fibers, especially those that undergo fermentation in the gut, help improve insulin sensitivity, regulate blood sugar levels, and reduce the risk of developing diabetes. Similarly, obesity, a global epidemic affecting approximately 30% of the population, is linked to an increased risk of CVD, diabetes, and other metabolic disorders (10). Research indicates that fiber consumption can aid in weight management by promoting satiety, improving gut microbiota composition, and modulating fat metabolism.

In addition to metabolic diseases, dietary fibers have also been implicated in the prevention and management of digestive system cancer, which are among the deadliest forms of cancer globally (11–13). Certain types of dietary fibers, such as pectin and resistant starch, have shown promise in reducing the risk of colon cancer by promoting beneficial gut bacteria and producing short-chain fatty acids (SCFAs) that help maintain a healthy colon environment. These fibers may also enhance the detoxification of carcinogens and inhibit the growth of cancer cells. Despite these promising findings, the precise relationship between fiber intake and cancer prevention remains an area of active research, with many questions yet to be answered (14).

Despite the growing body of evidence supporting the health benefits of dietary fiber, much remains unknown about the specific ways in which different types of fiber affect various diseases. Existing studies have largely focused on individual health conditions in isolation, but there is a need for more comprehensive research that examines how fiber may work synergistically to address multiple health issues at once. Furthermore, much of the current literature lacks clarity on the optimal types and amounts of fiber needed to achieve these health benefits.

This study aims to bridge these gaps by investigating the impact of dietary fibers on multiple chronic diseases, including CVDs, type II diabetes, obesity, and colon cancer. By examining how both soluble and insoluble fibers contribute to disease prevention and management, this research seeks to provide a deeper understanding of the mechanisms involved. The goal is to identify specific fiber types and dietary patterns that may offer the most promise in improving health outcomes and preventing disease progression.

In this study, a comprehensive review of the existing literature was conducted, including original research articles, review papers, and meta-analyses. Several studies were systematically reviewed to assess the current evidence on the role of dietary fiber in various health conditions This study will help clarify the role of dietary fiber not only as a digestive aid but as a powerful tool in the prevention and management of some of the world’s most prevalent and debilitating health conditions. By providing clearer insights on fiber intake, this research could help inform public health recommendations and clinical practices, ultimately contributing to better health at the population level.

### 1.1 Fiber-containing diets for CVD

As a group of conditions affecting the heart and blood vessels, CVD is one of the most important conditions in developed, developing and underdeveloped countries. CVD, including coronary disease (CHD), stroke, aortic atherosclerosis, and peripheral artery disease, have been identified as the first cause of mortality worldwide (15). CVD prevalence is increasing rapidly, therefore turning it into a global health concern and require more attention (16). It is estimated that 32% of deaths are related to cardiovascular diseases worldwide, which is approximately 17.9 million deaths per year (17). In overall, different factors determine the risk of CVD development, which can be divided into two groups: unmodifiable and modifiable factors. Gender, age and genetics are among unmodifiable factors. While, dietary habits are considered among modifiable factors that contribute to the development of CVD.

Dietary fiber is crucial for having a healthy diet for the cardiovascular system. Fiber-rich diets incorporation within the daily meals, can significantly improve overall well-being and heart health. Dietary fiber is categorized into two main groups: soluble fiber and insoluble fiber. Soluble fiber is dissolvable in water, forming a gel-like structure. This type of fiber is known for reducing LDL cholesterol, known as the bad cholesterol. Soluble fiber can bind to the bile acids within the digestive tract, extract them and lowers the cholesterol level in the blood. Studies have proved that the intake of soluble fiber can significantly reduce LDL as an important risk factor for coronary artery disease (18).

In research conducted by Ghavami et al. the effects of incorporating soluble fiber supplementation into adults’ diet and its potential influence of the level of blood lipid were studied. The aim was mainly to understand the impact of dietary fiber intake on overall cardiovascular health in the adult population. This study suggested that soluble fiber can in fact reduce the cardiovascular disease risk by managing the dyslipidemia (19).

There is a strong connection between the lifestyle and CVDs. A daily diet high in sugar and saturated fats is linked to CVD outbreaks. This diet also tends to be low in polyunsaturated fatty acids and fiber (20). In recent years, an expanding body of research has increasingly emphasized that following a consistent diet rich in fiber can significantly reduce the risk of developing CVD. These studies not only underline the importance of dietary fiber for overall health but also provide specific recommendations for daily intake. Experts suggest that adults should aim for a fiber consumption of 14 grams per 1,000 calories consumed. Additionally, they recommend that adult women strive for an intake of approximately 25 grams of fiber per day, while adult men should target around 38 grams daily. This focus on fiber-rich foods underscores their vital role in promoting heart health and preventing CVD (21).

A detailed comparison conducted across various meta-analyses revealed that the intake of dietary fiber has a substantial impact on the relative risk (RR) of mortality associated with CVD. When comparing people with the highest and lowest fiber intake, the data showed different risks for various health conditions. For overall heart disease, the risk ranged from 0.72 to 0.91. For stroke, it was between 0.83 and 0.93, and for coronary heart disease, the risk ranged from 0.76 to 0.93. These results suggest that eating more fiber may lower the risk of dying from these conditions (22).

Also, some studies imply that the source of the fiber is more important than its amount of intake (23). Fibers from different sources can exhibit different physiological effects. For instance, soluble fiber has a cholesterol-lowering effect, while insoluble fiber has an interaction with intestinal food absorption and reduces the clotting factors (24, 25).

The exact ways in which dietary fibers contribute to a reduced risk of CVD remain somewhat ambiguous. However, studies have demonstrated that both soluble and insoluble fibers play significant roles in various bodily functions. One key effect of fiber consumption is the enhancement of gastric distension, which occurs when the stomach expands as it fills with food. This process triggers the release of certain hormones from the gut that promote a sense of fullness, known as satiety. Consequently, individuals often find themselves eating less overall. Adopting a diet abundant in fiber not only helps to regulate appetite but also encourages healthier eating habits over time. As people consume less food due to increased feelings of fullness, they are likely to experience weight loss. This reduction in body weight can have profound effects on metabolic health, including better regulation of glucose levels.

Improved glucose metabolism is crucial for preventing conditions such as insulin resistance, which can lead to further cardiovascular complications. In summary, the interplay of enhanced satiety, weight management, and improved metabolic function illustrates how a diet high in fiber can play a vital role in lowering the risk of cardiovascular disease. By making fiber a priority in their diets, individuals may significantly improve their overall health and longevity (26).

In a comprehensive study led by Ebab S. et al., the researchers explored the relationship between dietary fiber intake and mortality rates from coronary heart disease. Their findings indicated that consuming both soluble and insoluble fibers is associated with a significant reduction in the risk of death linked to this condition. What is particularly noteworthy is the suggestion that fibers specifically sourced from fruits and cereals may offer even more substantial protective effects against CVD. This insight underscores the importance of incorporating a variety of fiber-rich foods into one’s diet, as different types of fiber can have unique benefits for heart health. Fruits and whole grains are not only rich in fiber but also packed with essential vitamins, minerals, and antioxidants, which collectively contribute to better cardiovascular health. By emphasizing these food sources, individuals may enhance their dietary strategies aimed at reducing the risk of CVD. Moreover, the study implies that dietary patterns that prioritize fruit and cereal fibers could lead to improved overall well-being. As part of a balanced diet, these fibers may aid in regulating blood sugar levels, lowering cholesterol, and promoting a healthy weight, all crucial factors in mitigating the risk of heart disease. This highlights the potential for targeted dietary interventions that focus on increasing the intake of specific fiber types to improve cardiovascular outcomes (27).

Overall, it can be concluded that the maintaining a diet containing high levels of fiber, can significantly improve hearth condition and be beneficial for CVD. This section is summarized in Table 1.

**Table 1:** Key points of fiber-rich diet influence on CVD.

Topic	Key points
Cardiovascular Diseases (CVD)	- Leading cause of death worldwide (32% of global deaths, ~17.9 million annually) - Includes coronary heart disease (CHD), stroke, aortic atherosclerosis, and peripheral artery disease. - Prevalence rising globally, posing a significant health concern.
Risk Factors for CVD	- Unmodifiable: Gender, age, genetics - Modifiable: Dietary habits (e.g., fiber intake)
Role of Dietary Fiber in Heart Health	- Essential for cardiovascular health - Soluble fiber: Lowers LDL cholesterol (bad cholesterol) by binding to bile acids and reducing cholesterol levels. - Insoluble fiber: Reduces clotting factors and aids intestinal absorption.
Soluble Fiber and CVD Risk	- Soluble fiber reduces LDL cholesterol and helps manage blood lipids, reducing CVD risk (studies show improvement in dyslipidemia). - Daily intake recommendations: 14 g fiber per 1,000 calories (women: 25 g, men: 38 g).
Effect of Fiber on Mortality Risk	- High fiber intake linked to lower mortality from CVD: - Overall CVD: RR 0.72–0.91 - Stroke: RR 0.83–0.93 - Coronary heart disease: RR 0.76–0.93
Importance of Fiber Source	- Fiber source may be more important than quantity. - Soluble fiber: Reduces cholesterol. - Insoluble fiber: Improves intestinal health, reduces clotting factors.
Additional Benefits of Fiber	- Enhances satiety, helps with weight loss, and improves metabolic health (e.g., glucose regulation). - Helps regulate appetite, leading to lower food intake and improved weight management, reducing CVD risk factors.
Study Findings	- Studies show a connection between fiber intake and lower CVD-related mortality, especially with fibers from fruits and cereals. - Fruits and whole grains provide fiber plus essential vitamins, minerals, and antioxidants, which further support heart health.
Conclusion	- A high-fiber diet significantly improves heart health and reduces CVD risk.

### 1.2 Fiber-containing diets in type II diabetes management

Type II diabetes and its many side effects, are among the most widespread health issues across the globe, with the number of affected individuals increasing daily (28). An essential and effective approach to managing this condition, as well as improving HbA1c levels, is through careful dietary management. Embracing a low-calorie diet that is abundant in vitamins, fiber, and minerals can help maintain stable blood sugar levels. Limiting or completely eliminating sugar and processed foods also supports healthy lipid profiles.

A well-structured diet not only aids in metabolic regulation but also promotes overall health. Prioritizing whole, nutrient-rich foods allows individuals to better control their blood glucose levels, which is critical for effective diabetes management. Foods high in fiber, such as fruits, vegetables, whole grains, and legumes, are particularly advantageous because fiber slows down digestion and enhances insulin sensitivity. This dietary strategy not only assists in blood sugar control but also supports cardiovascular health and reduces the risk of complications linked to diabetes. Managing Type II diabetes through thoughtful dietary choices can lead to improved health outcomes and a higher quality of life for those affected by this chronic condition. By making informed decisions about food, individuals can take significant steps toward better diabetes management and overall well-being (29).

Dietary fibers are significantly important in a healthy diet. These fibers contain a vary of complex molecules based on saccharide, which can bind potential nutrients and their precursors, leading to absorption limitation. By food ingestion modulation, dietary fibers can reduce the risk of hyperglycemia, hyperlipidemia, and hypercholesterolemia. Dietary fiber can lower the risk of CVD, colon cancer and also, type II diabetes mellitus (27). Studies have indicated that an intake of 50 g of dietary fiber every day, can enhance the metabolism of glycolipid in diabetic patients (30).

Dietary fibers have shown significant promise in their ability to reduce the risk of developing Type II diabetes. The effectiveness of this risk reduction appears to be closely linked to both the specific type of fiber consumed and the quantity ingested. Dietary fibers are increasingly recognized for their potential role in reducing the risk of Type II diabetes. The effectiveness of this protective effect seems to be influenced by both the type of fiber and the amount consumed.

A study conducted by Wang et al. highlighted the impact of soluble dietary fibers on Type II diabetes biomarkers in mouse models. Their research revealed that incorporating soluble fibers into a high-fat diet led to significant improvements in insulin sensitivity. This is particularly important, as enhanced insulin sensitivity helps regulate blood sugar levels and can prevent the onset of diabetes. Additionally, the inclusion of dietary fibers in the diet was linked to reduced weight gain. This suggests that these fibers can help mitigate some of the negative effects associated with high-fat diets. These findings indicate that not all fibers are equal in effect; soluble fibers may offer more substantial benefits for metabolic health. This opens up new possibilities for dietary interventions aimed at lowering the risk of diabetes and improving overall health (31).

Soluble dietary fibers have been shown to effectively lower lipid and glucose levels in individuals with Type II diabetes mellitus. Recent research has highlighted galactomannans as the most beneficial type of soluble fiber for these patients. This particular fiber has been linked to significant reductions in important health indicators, including HbA1c, triglycerides, total cholesterol, fasting blood glucose, and LDL cholesterol. In addition to galactomannans, other soluble fibers such as xylo-oligosaccharides and gum Arabic have also proven effective in improving cholesterol levels. These fibers contribute to lowering total cholesterol and HDL cholesterol, further underscoring the diverse benefits of soluble fibers in managing metabolic health. Incorporating these types of fibers into daily diets can provide crucial support for individuals striving to manage their diabetes.

By emphasizing fiber-rich foods, patients can enhance their ability to maintain stable blood sugar levels. This ultimately improves their overall well-being (32). Recent research has established a compelling link between grain-based dietary fibers and a lower risk of developing diabetes. The findings suggest that these fibers may be more effective than those derived from other sources. Studies indicate that the unique composition of fibers found in whole grains plays a significant role in improving insulin sensitivity and regulating blood sugar levels. Incorporating grain-based fibers into the diet appears to provide enhanced benefits for metabolic health, making them a valuable component for individuals looking to manage or reduce their risk of diabetes. This highlights the importance of selecting whole grain foods as a primary source of dietary fiber for optimal health outcomes. By focusing on these fiber-rich grains, individuals can take important steps toward better blood sugar control and overall well-being (33).

Type II diabetes is closely associated with the rising prevalence of obesity. This condition typically arises from insulin resistance and/or impaired insulin secretion from the beta cells in the pancreas. These dysfunctions disrupt the normal metabolism of proteins, lipids, and carbohydrates, leading to a range of metabolic disturbances. As the body becomes less responsive to insulin, or as the pancreas fails to produce adequate insulin, the regulation of blood sugar levels becomes compromised. This not only contributes to hyperglycemia but also affects the metabolism of fats and proteins, further exacerbating the metabolic imbalances associated with obesity. Understanding this relationship is crucial for developing effective strategies for the prevention and management of Type II diabetes. This is especially important in the context of weight management and lifestyle interventions (34).

Insulin resistance is a significant marker of a prediabetic condition and can ultimately lead to the onset of Type II diabetes. This phenomenon occurs when the body’s insulin is unable to effectively lower blood glucose levels, even when there is a sufficient supply of insulin present in the bloodstream. Consequently, the body responds by elevating the fasting insulin levels in the plasma. If this state persists, it can result in hyperinsulinemia, characterized by abnormally high insulin levels. This ongoing imbalance can disrupt normal metabolic functions and increase the risk of developing more severe health complications associated with diabetes. It highlights the importance of early intervention to manage insulin resistance and prevent the progression to Type II diabetes (35).

Soluble dietary fiber found in specific fruits and vegetables can enhance the way macronutrients are absorbed by forming gels in the digestive system. This gel-forming property slows down digestion, which in turn helps to mitigate the rise in blood glucose levels following a meal. By reducing the postprandial glucose response, these fibers play a crucial role in maintaining stable blood sugar levels and supporting overall metabolic health (36).

Research has shown that consuming soluble fiber can enhance insulin sensitivity and lower postprandial blood glucose levels in both diabetic and non-diabetic individuals. This improvement in insulin response helps the body manage blood sugar more effectively after meals, contributing to better overall metabolic health. By incorporating soluble fiber into their diets, people can experience beneficial effects on glucose regulation, regardless of their diabetic status (37). Additionally, the consumption of insoluble fiber has been shown to significantly improve insulin sensitivity in both diabetic and non-diabetic individuals. This type of fiber contributes to better metabolic function by enhancing the body’s ability to respond to insulin, thereby promoting more effective blood sugar regulation. By including insoluble fiber in their diets, individuals can support their overall health and potentially reduce the risk of developing insulin resistance (38).

Dietary fibers play a vital role in enhancing the production of short-chain fatty acids (SCFAs) during the digestive process. These SCFAs, which are produced when fiber is fermented by gut bacteria, have a wide array of beneficial effects on metabolic health. One significant outcome of SCFA production is the stimulation of leptin release from adipose tissue. Leptin is a hormone that helps regulate energy balance and appetite, signaling the body to reduce food intake.

Additionally, SCFAs promote the secretion of other important hormones, including peptide YY and glucagon-like peptide-1 (GLP-1). Peptide YY is involved in appetite suppression, while GLP-1 enhances insulin secretion and inhibits glucagon release, both of which are critical for maintaining stable blood sugar levels. This intricate hormonal interplay is essential for effective appetite control and metabolic regulation. Furthermore, SCFAs have been found to improve insulin sensitivity, a key factor in managing blood glucose levels, particularly in individuals with Type II diabetes. By enhancing the body’s response to insulin, SCFAs help facilitate the uptake of glucose into cells, thereby lowering blood sugar levels after meals. In summary, the fermentation of dietary fibers into short-chain fatty acids not only supports the release of hormones that regulate hunger and glucose metabolism but also enhances insulin sensitivity. This multifaceted approach makes dietary fiber an important component in the dietary management of Type II diabetes and overall metabolic health. By incorporating a variety of fiber-rich foods into their diets, individuals can promote the production of SCFAs and support their metabolic well-being (39). The key points of this section are categorized in Table 2.

**Table 2:** Key points of fiber-rich diet influence on type II diabetes.

Topic	Key points
Type II Diabetes Management	- A low-calorie diet with vitamins, fiber, and minerals supports HbA1c control and stable blood sugar levels. - Avoid sugar and processed foods for better metabolic health. - Food rich in fiber (fruits, vegetables, whole grains, legumes) improve insulin sensitivity and cardiovascular health.
Role of Dietary Fiber	- Dietary fibers reduce risk of hyperglycemia, hyperlipidemia, and hypercholesterolemia. - A 50 g/day fiber intake improves glycolipid metabolism in diabetics.
Types of Fiber and Their Impact	- Soluble fibers (e.g., galactomannans, gum Arabic) lower HbA1c, triglycerides, total cholesterol, fasting glucose, and LDL cholesterol. - Grain-based fibers are particularly effective in improving insulin sensitivity and regulating blood sugar.
Obesity and Insulin Resistance	- Obesity contributes to insulin resistance, disrupting metabolism of proteins, lipids, and carbohydrates. - Insulin resistance is a precursor to Type II diabetes. High insulin levels can lead to hyperinsulinemia, worsening metabolic imbalances.
Soluble Fiber Benefits	- Soluble fiber slows digestion, reducing post-meal glucose spikes. - Improves insulin sensitivity and blood glucose management in both diabetic and non-diabetic individuals.
Insoluble Fiber Benefits	- Insoluble fiber improves insulin sensitivity and metabolic function.
Short-Chain Fatty Acids (SCFAs)	- SCFAs from fiber fermentation enhance insulin sensitivity, promote leptin release (reducing appetite), and support GLP-1 and peptide YY secretion. - SCFAs aid in better glucose uptake, lowering blood sugar levels after meals.

### 1.3 Fiber-containing diets for obesity

Obesity and its related complications, such as Type II diabetes mellitus, hypertension, and dyslipidemia, represent significant challenges to public health. The growing prevalence of obesity is associated with numerous health issues that not only affect individual quality of life but also place considerable demands on healthcare systems. The rise in obesity can be attributed to various factors, including unhealthy eating patterns, lack of physical activity, and environmental influences. As body weight increases, the likelihood of developing associated health problems also escalates. Type II diabetes often emerges as a direct result of insulin resistance, a condition commonly seen in individuals with obesity (40). This metabolic disorder can lead to serious health complications, including heart disease, kidney issues, and nerve damage (41).

Hypertension, or elevated blood pressure, frequently coexists with obesity (42). The excess weight puts additional strain on the cardiovascular system, which can lead to sustained increases in blood pressure. This, in turn, heightens the risk of heart attacks and strokes. Dyslipidemia, characterized by abnormal lipid levels in the bloodstream, is also common among those with obesity. This condition can result in elevated cholesterol and triglyceride levels, further increasing the risk of cardiovascular problems. Effectively addressing obesity and its associated conditions requires a comprehensive strategy that promotes healthier dietary choices, encourages regular physical activity, and implements supportive community and policy initiatives. By focusing on these areas, we can enhance public health and work toward reducing the incidence of chronic diseases linked to obesity (43).

Obesity is recognized as a medical condition that significantly increases the risk of various serious health issues, including CVD and certain cancers. It is estimated that obesity is linked to more than 2.8 million deaths globally each year. This staggering figure highlights the critical importance of addressing obesity in public health efforts. Given its profound effects on health, tackling obesity is essential not only for individual health outcomes but also for alleviating the strain on healthcare systems. Developing effective prevention and intervention strategies is vital for promoting healthier lifestyles and reducing the prevalence of obesity-related diseases (44).

To effectively prevent and manage obesity, dietary interventions focused on increasing the intake of fiber-rich foods are highly recommended. Incorporating a variety of vegetables, whole grains, and fruits into one’s diet can significantly contribute to weight management. These foods are not only low in calories but also high in essential nutrients and fiber, which promotes satiety and helps regulate appetite. By emphasizing these dietary changes, individuals can foster healthier eating habits that support weight control and enhance their overall well-being. Adopting a diet rich in fiber can be a sustainable strategy for achieving and maintaining a healthy weight while also reducing the risk of obesity-related health issues (45, 46).

The potential mechanisms by which dietary fibers help manage obesity are quite diverse. One important mechanism is their ability to improve insulin sensitivity, which enhances the body’s responsiveness to insulin and helps maintain stable blood sugar levels. This effect can prevent significant glucose spikes after eating, thereby reducing the risk of developing insulin resistance. In addition, dietary fibers contribute to the regulation of lipid levels in the body. They support the maintenance of healthy cholesterol levels and help manage triglycerides, which are crucial for overall metabolic health. Moreover, dietary fibers can decrease the absorption of cholesterol from food during digestion. By limiting the amount of cholesterol that enters the bloodstream, fibers can play a role in reducing the risk of cardiovascular diseases and other related conditions (47).

It’s noteworthy that dietary fibers play a crucial role as substrates for the anaerobic fermentation carried out by gut microbiota. This fermentation process results in the production of key metabolites, such as propionate, butyrate, and acetate. SCFAs are essential for promoting gut health and supporting various metabolic functions. For example, butyrate is particularly beneficial for the colon, providing energy to its cells and exhibiting anti-inflammatory effects. Meanwhile, propionate and acetate contribute to important physiological processes, including improved insulin sensitivity and appetite regulation. By fostering the growth of beneficial gut bacteria and facilitating the production of these metabolites, dietary fibers help maintain a healthy gut microbiome. This underscores the importance of including fiber-rich foods in the diet to enhance both digestive health and overall metabolic well-being (48).

The impact of various dietary fibers on weight control and overall health remains a subject of debate among researchers. A recent study by Zheng et al. delved into this topic and yielded significant findings. The results demonstrated that all three types of dietary fibers (soluble, insoluble, and total fiber) extracted from the same source effectively reduced body weight in obese mice. These findings highlight that increasing fiber intake, regardless of its type, can play a crucial role in weight management. This research contributes to our understanding of how different fibers can be leveraged in dietary approaches aimed at promoting weight loss and enhancing metabolic health. Further studies are necessary to explore the underlying mechanisms and to assess the applicability of these results in human populations (33).

Research has consistently shown that diets that are high in both fiber and protein can play a significant role in promoting effective weight management and enhancing metabolic health. A study conducted by Erin L et al. specifically examined this relationship, providing valuable insights into how these dietary components interact. The study revealed that participants who consumed a multi-ingredient nutritional shake rich in protein and fiber experienced noticeable weight loss. Not only did these individuals shed pounds, but they also demonstrated significant reductions in body fat and LDL cholesterol levels, which are important indicators of cardiovascular health. Additionally, there was a marked increase in adiponectin levels, a hormone that plays a crucial role in regulating glucose levels and fatty acid breakdown.

Higher adiponectin levels are often associated with lower risks of obesity-related conditions. Interestingly, the study also noted that subjects who consumed shakes with lower amounts of protein and fiber exhibited some positive health changes as well. However, the extent of these changes was less pronounced compared to those who included higher levels of both nutrients in their diets. This finding underscores the potential benefits of prioritizing fiber and protein in dietary choices. The results from Erin L et al.’s study highlight the importance of multi-ingredient nutritional interventions that emphasize both fiber and protein, suggesting that such diets can lead to more substantial improvements in weight loss and metabolic health. This indicates a promising avenue for individuals looking to enhance their overall well-being through dietary modifications. Further research is needed to explore the long-term effects of high-fiber and high-protein diets, as well as their implications for various populations (49).

As a conclusion to this study, it can be determined that a consuming high amount of fiber and protein as a shake before breakfast and lunch, can positively influence metabolic outcomes in overweight adult individuals and help with weight loss. Additionally, consuming a fat-, fiber-, and protein-containing preload before main meals can be considered a strategy to enhance satiety and decrease postprandial hyperglycemia. This approach promotes weight loss and reduces the risk of Type II diabetes.

Research indicates a significant link between the development of obesity and the characteristics of the gut microbiome. Individuals with obesity often display a unique gut microbiome that varies in terms of diversity, function, and composition compared to those with a healthier weight. This altered microbiome can influence metabolic processes, affecting how the body extracts energy from food and regulates fat storage (50, 51). For instance, specific bacterial populations may become more prevalent in obese individuals, leading to changes in fermentation processes and short-chain fatty acid production. These differences can contribute to inflammation and insulin resistance, further exacerbating weight gain and metabolic disorders. Moreover, the reduced diversity of gut bacteria often seen in obese individuals may diminish the microbiome’s ability to perform essential functions, such as metabolizing fiber and supporting immune responses. This highlights the importance of maintaining a balanced gut microbiome for overall health and weight management.

Understanding the complex interactions between gut microbiota and obesity can pave the way for new dietary and therapeutic strategies aimed at restoring microbiome balance and promoting weight loss. Further studies are essential to explore the underlying mechanisms and potential interventions to improve metabolic health through microbiome modulation (52). Thus, targeting the gut microbiome emerges as a promising strategy for both preventing and treating obesity. One effective way to achieve this is by increasing dietary fiber intake. Since fiber is indigestible by the human body, it reaches the intestines where it can be fermented by gut bacteria. This fermentation process produces SCFAs, which are beneficial metabolites. SCFAs have a variety of positive effects on energy metabolism and maintaining metabolic balance. They play a role in regulating appetite, influencing fat storage, and reducing inflammation. By enhancing these metabolic processes, SCFAs can support effective weight management and potentially prevent obesity. Additionally, a diet rich in fiber fosters a diverse gut microbiome, which is crucial for overall health. This diversity helps promote the production of beneficial compounds, further strengthening the connection between gut health and weight control. Therefore, incorporating more fiber-rich foods into daily meals could serve as an effective approach for individuals aiming to manage their weight. It also helps enhance their metabolic health. As ongoing research explores these connections, targeting the gut microbiome through dietary changes may provide valuable insights in the fight against obesity (53).

Research has indicated that nuts may play a significant role in reducing the risk of CVD and Type II diabetes. Numerous studies suggest that incorporating nuts into one’s diet can lead to positive outcomes for both heart health and metabolic regulation.

Nuts are packed with beneficial fats, particularly monounsaturated and polyunsaturated types, which are known to improve cholesterol profiles and reduce inflammation in the body. These effects are essential for maintaining a healthy cardiovascular system. Moreover, nuts are rich in vital nutrients such as fiber, vitamins, and minerals, which contribute to overall health. In addition to their heart-healthy properties, nuts have been associated with improved blood sugar control, an important factor in preventing and managing Type II diabetes. The fiber found in nuts aids in stabilizing blood sugar levels, while their healthy fats can enhance insulin sensitivity.

By including a variety of nuts in a balanced diet, individuals can take proactive steps to support heart health and mitigate the risk of diabetes, making them an excellent choice for those looking to improve their overall dietary habits (54). Due to their high satiety properties, nuts can effectively help control appetite after meals. Consuming nuts can significantly reduce feelings of hunger and curb the urge to snack further. Their unique combination of healthy fats, protein, and fiber contributes to a lasting sense of fullness, making it easier to regulate overall food intake. By providing a satisfying snack option, nuts can help diminish cravings and prevent overeating, which is particularly beneficial for those aiming to maintain a healthy weight or improve their eating patterns. Incorporating nuts into meals or enjoying them as snacks can promote a feeling of satisfaction, reducing the likelihood of opting for less nutritious choices later in the day (55).

Although nuts are well-known for their high insoluble fiber content and other beneficial nutrients, they are frequently left out of traditional weight-loss plans due to their significant caloric density. This raises important questions about how nuts can fit into effective weight management strategies. To explore this topic, a study conducted by S. Petersen et al. examined the impact of incorporating peanuts into a weight-loss regimen. The results showed that participants who consumed 35 grams of peanuts before their two main meals each day experienced weight loss comparable to those following a standard low-fat diet. This finding is particularly significant, suggesting that peanuts can be part of a weight management approach without hindering weight loss efforts. The study also evaluated various metabolic markers to assess overall health. No notable differences were found in key indicators, including HbA1c, fasting blood glucose, 2 h postprandial glucose, and fasting insulin levels, between the peanut-inclusive diet and the traditional low-fat diet. This implies that including peanuts does not negatively impact blood sugar control, which is crucial for individuals managing diabetes or prediabetes.

Additionally, one of the standout findings was the more significant reduction in systolic blood pressure among those who included peanuts in their weight-loss diet. Lowering systolic blood pressure is vital, as it is associated with a decreased risk of CVD. This suggests that peanuts not only serve as a satisfying snack but may also contribute positively to heart health. Overall, these findings support the idea that incorporating nuts into a weight management plan can be beneficial. They provide essential nutrients and help promote feelings of fullness while supporting weight loss goals. By enhancing overall metabolic health and potentially benefiting cardiovascular well-being, nuts can be a valuable addition to a balanced diet aimed at maintaining a healthy weight (56). The key points of this section are summarized in Table 3.

**Table 3:** Key points of fiber-rich diet influence on obesity.

Topic	Key points
Obesity and Related Health Risks	- Obesity increases risk of Type II diabetes, hypertension, dyslipidemia, cardiovascular disease, and metabolic issues.
Role of Dietary Fiber	- Fiber (from vegetables, whole grains, fruits) aids weight management, improves insulin sensitivity, and supports heart health by regulating cholesterol and blood sugar.
Gut Health and SCFAs	- Fiber fermentation produces SCFAs (butyrate, propionate, acetate), improving insulin sensitivity, reducing inflammation, and regulating appetite.
High-Fiber and Protein Diets	- Diets rich in fiber and protein help with weight loss, reduce body fat, and improve metabolic health, including lowering blood sugar and cholesterol.
Nuts and Weight Management	- Nuts (like peanuts) improve cholesterol, reduce inflammation, enhance satiety, and aid weight loss, without negatively impacting blood sugar control.

### 1.4 Fiber-containing diets for colon-cancer prevent and treatment

Cancer is one of the world’s most occurring and deadly conditions. Many studies are devoted to analyzing, prevention, and management of cancer (57). Colon cancer ranks as the third most frequently diagnosed cancer across the globe and is associated with a high risk of death. Current estimates indicate that, if the trends persist, about 50% of individuals in Western nations may experience at least one colorectal tumor by the time they reach 70 years old. This unsettling statistic highlights the urgent necessity for effective prevention strategies and early detection methods. The growing prevalence of colon cancer underscores the importance of raising awareness and implementing proactive health measures to diminish its impact on public health (58, 59).

The two primary contributors to the onset of colon cancer are genetic predispositions and environmental factors, particularly dietary choices. Research indicates that approximately 47% of colorectal cancer cases might be preventable through the adoption of a healthier lifestyle. This underscores the significance of making conscious decisions regarding nutrition and physical activity, as these factors can substantially influence an individual’s risk of developing this disease. By prioritizing healthy habits, individuals can actively work toward reducing their chances of colon cancer (60).

Dietary fibers, such as pectin, lignin, oligosaccharides, non-starch polysaccharides, and similar compounds, are significantly associated with various health benefits. These fibers are essential for maintaining digestive health, as they aid in promoting regular bowel movements and fostering a balanced gut microbiome. Furthermore, they can help manage blood sugar levels, reduce cholesterol, and enhance satiety, which may support effective weight control. By including a diverse range of fiber types in one’s diet, individuals can improve their overall health and lower the risk of developing chronic conditions.

Both soluble and insoluble dietary fibers are recognized for their potential in reducing the risk of colorectal cancer. A study conducted by Arayici et al. explored the effectiveness of these fiber types, along with total fiber, in this context. The researchers found that the impact of soluble, insoluble, and total fiber on colorectal cancer prevention was remarkably similar. In their analysis, the effect size (ES) for soluble fiber was calculated to be 0.78, with a 95% confidence interval spanning from 0.66 to 0.92. This suggests a strong association between soluble fiber intake and a reduced risk of colorectal cancer. In comparison, the effect size for insoluble fiber was 0.77, indicating it also provides significant protective benefits. Total fiber showed an effect size of 0.75, which further supports the notion that incorporating a variety of fiber types into one’s diet is beneficial. These results highlight the importance of both soluble and insoluble fibers in a healthful diet aimed at preventing colorectal cancer. By consuming a diverse array of fiber-rich foods, individuals can maximize their protective effects against this disease. This study not only reinforces the need for dietary fiber in cancer prevention strategies but also encourages further research into how different types of fiber interact with gut health and overall wellness. The findings advocate for public health recommendations that emphasize increasing fiber intake as part of a balanced diet to promote long-term health and reduce cancer risk (61).

Extensive research has investigated how dietary fibers may contribute to a reduced risk of colorectal cancer. One significant mechanism proposed is that dietary fibers decrease the amount of time food spends in contact with potential carcinogens in the intestinal lumen. This shorter exposure could help lessen the harmful effects associated with these substances.

In addition to this, dietary fibers are known to foster the growth of beneficial gut microbiota. A healthy microbiome is essential for effective digestion and overall health, as it enhances the body’s metabolic processes. Additionally, it can produce beneficial compounds that may help protect against cancer. Furthermore, dietary fibers can influence the host’s metabolism by modulating various biological functions. They assist in the regulation of bile acids and promote the fermentation process, leading to the production of short-chain fatty acids, which have been shown to offer protective benefits for the colon. These interconnected mechanisms highlight the critical role of dietary fibers in maintaining gut health and reducing the risk of colorectal cancer.

As ongoing research sheds more light on these relationships, it becomes increasingly clear that incorporating fiber-rich foods into one’s diet is a valuable strategy for cancer prevention and overall well-being (62). In research conducted by Hidaka et al., a correlation was identified between higher fruit consumption and a decreased risk of tumors characterized by BRAF mutations. The study highlighted that increased fiber intake also appears to lower the risk of colorectal cancer, particularly in cases marked by specific genetic alterations such as microsatellite instability-high (MSI-high), CpG island methylator phenotype-positive (CIMP-positive), and mutations in both KRAS and BRAF genes. These results emphasize the protective role that fruit and fiber-rich diets can play in reducing the likelihood of developing certain types of colorectal cancer. By focusing on dietary patterns that include ample amounts of fruits and fibers, individuals may actively contribute to lowering their risk associated with these genetic factors. This research adds to the growing body of evidence that underscores the importance of nutrition in cancer prevention efforts (63).

A variety of natural polysaccharides, including lignin, pectin, alginate, inulin, and gums, have been the focus of research for their potential anti-tumor properties. These fiber-based polysaccharides demonstrate promise not only in their ability to inhibit tumor growth but also in their applications for drug delivery in cancer therapies. Their unique structural characteristics enable them to effectively encapsulate therapeutic agents, improving the targeted delivery of drugs to tumor sites while minimizing side effects on healthy tissues. By leveraging the inherent properties of these polysaccharides, researchers are exploring innovative ways to enhance the efficacy of cancer treatments. Their roles in modulating the immune response and supporting gut health further highlight their multifaceted potential in oncological applications. As studies continue to unfold, these natural compounds could become valuable components in developing more effective and safer cancer treatment strategies (64).

Dietary fibers can serve as effective encapsulating agents for oral drug delivery specifically targeting the colorectal region, thanks to their fermentable characteristics. These fibers can encapsulate therapeutic compounds, ensuring that they remain intact during transit through the gastrointestinal tract. Once they reach the colon, the fermentation process activates the release of the encapsulated drugs at the desired site. This targeted delivery approach not only enhances the bioavailability of medications but also minimizes systemic side effects, making it a promising strategy for treating colorectal diseases. By utilizing the natural properties of dietary fibers, researchers are paving the way for innovative drug formulations that could improve therapeutic outcomes for patients while leveraging the health benefits of fiber itself. As research in this area progresses, these fiber-based delivery systems may become a vital component in the future of colorectal health treatments (65).

Whole grains, known for their high fiber content, have demonstrated a significant capacity to lower the risk of colorectal cancer. Research has revealed an inverse relationship between whole grain consumption and the likelihood of developing colorectal cancer, with particularly strong associations noted for rectal cancer and distal tumors. This protective effect is likely attributed to the dietary fiber found in whole grains, which aids in promoting healthy digestion and supporting a balanced gut microbiome. Additionally, whole grains contain various bioactive compounds, such as antioxidants and phytonutrients, that may further contribute to their cancer-preventive properties. By incorporating whole grains into one’s diet, individuals may not only enhance their overall nutrition but also take proactive steps toward reducing their risk of colorectal cancer. As awareness of these benefits grows, whole grains can play a vital role in dietary strategies aimed at improving colorectal health (66).

In another study, it was shown that a daily intake of 90 grams of refined grains could result in a 6% decrease in the overall cancer risk. This finding indicates that while whole grains are frequently praised for their health benefits, refined grains may also have a role in cancer prevention when consumed appropriately. This research highlights the nuanced relationship between diet and health, suggesting that certain refined grain products might contribute positively to cancer risk reduction as part of a varied diet. However, it remains vital to view these findings within the larger framework of dietary habits, emphasizing the significance of including whole grains, fruits, and vegetables to foster long-term health and reduce disease risk. As further studies are conducted, a clearer understanding of how different types of grains affect cancer prevention will emerge (67). Other studies have revealed a negative association between whole grain consumption and the risk of several cancers, including colorectal, gastric, and esophageal cancers. Specifically, findings indicate that increased intake of whole grains is linked to a decreased risk of colorectal cancer (with a 95% confidence interval of 0.84–0.93), gastric cancer (95% confidence interval of 0.53–0.79), and esophageal cancer (95% confidence interval of 0.44–0.67). These results emphasize the potential protective benefits of whole grains, suggesting that they may play a significant role in cancer prevention. The advantageous components found in whole grains, such as dietary fiber, antioxidants, and a variety of phytonutrients, are likely contributing factors to these protective effects. As research continues to support these findings, incorporating whole grains into the diet can be a valuable approach to promote health and lower cancer risk (68).

Consequently, it is advisable to enhance the daily intake of whole grains as part of a balanced diet. Incorporating a variety of whole grain foods can provide essential nutrients, promote digestive health, and potentially lower the risk of certain cancers. Making whole grains a staple in meals can contribute to overall well-being and serve as a proactive measure for better health. The key points of this section are summarized in Table 4.

**Table 4:** Key points of fiber-rich diet influence on colon cancer prevention.

Topic	Key points
Colon Cancer	- 50% of Western adults may develop colon cancer by age 70; 47% of cases are preventable through lifestyle changes.
Fiber and Prevention	- Both soluble and insoluble fiber reduce colon cancer risk by improving gut health and reducing carcinogen exposure.
Fruit & Genetic Protection	- High fiber, especially from fruits, lowers cancer risk, particularly with genetic factors like BRAF mutations.
Whole Grains	- Whole grains lower colorectal cancer risk, with fiber and antioxidants playing key roles.
Refined Grains	- Moderate refined grain intake may slightly reduce cancer risk, but whole grains offer more protection.
Cancer Risk Reduction	- Increased whole grain intake reduces risk of colorectal, gastric, and esophageal cancers.

### 1.5 Fiber-containing diets for inflammatory diseases

Chronic inflammatory diseases represent a category of non-infectious conditions where prolonged inflammation is a central factor. This ongoing inflammatory response is linked to an elevated risk of several serious health issues, including CVD, type II diabetes, and metabolic syndrome. Each of these conditions not only poses significant health threats but also negatively impacts overall quality of life and reduces longevity. The relationship between chronic inflammation and these diseases is complex. For instance, inflammation can disrupt normal metabolic processes, leading to insulin resistance and lipid abnormalities, which are key contributors to type II diabetes and heart disease. Furthermore, chronic inflammation can promote arterial damage and plaque buildup, heightening the risk of heart attacks and strokes. Given this connection, reducing inflammation becomes essential in lowering the risk of CVD and other related conditions. Strategies to combat inflammation include adopting an anti-inflammatory diet rich in whole foods, such as fruits, vegetables, whole grains, and healthy fats. Regular physical activity is also beneficial, as it can help regulate inflammatory markers in the body. Additionally, managing stress through mindfulness practices and adequate sleep can further support inflammation reduction. Understanding the critical role of inflammation in chronic diseases highlights the importance of proactive health measures in the fight against these prevalent health issues (69).

By prioritizing inflammation management through these lifestyle changes, individuals can improve their health outcomes and enhance their quality of life, potentially leading to a longer, healthier life. Adopting healthier lifestyle choices is essential for reducing inflammation and enhancing overall well-being. Key actions include quitting smoking, boosting physical activity, and improving dietary habits. Evidence indicates that diets high in fiber, particularly those rich in fruits and vegetables, can effectively lower inflammation levels and promote better health.

A fiber-rich diet is beneficial because it provides vital nutrients and supports proper digestion, both of which can help decrease inflammatory responses in the body. Fruits and vegetables are particularly valuable due to their high content of antioxidants and phytochemicals, which help counteract oxidative stress, a significant factor in chronic inflammation. Additionally, these foods contain prebiotic fibers that nurture beneficial gut bacteria, further enhancing gut health and metabolic efficiency. Regular exercise is another crucial component in the fight against inflammation. Engaging in physical activity has been shown to reduce levels of inflammatory markers in the body, contributing to a healthier inflammatory balance. When combined with a diet high in fiber, these lifestyle changes can create a powerful synergy that supports better health outcomes. These changes not only lead to immediate health improvements but also establish a foundation for long-lasting wellness (70, 71).

Ma et al. found that consuming higher amounts of dietary fiber is linked to alterations in gut microbiome composition, which can significantly decrease the risk of inflammatory diseases like CVD and inflammatory bowel disease. This beneficial impact was particularly noted with fiber sourced from fruits. The increase in fruit fiber intake seems to promote the growth of advantageous gut bacteria that are vital for regulating inflammation. These microbial changes can enhance gut health and improve overall metabolic processes, leading to a lower likelihood of developing inflammatory conditions. This research highlights the value of including fiber-rich foods, especially those from fruit, in the diet to foster a healthy microbiome and reduce the risk of various inflammatory diseases (72).

Additionally, research has shown that maintaining a low-fat, high-fiber diet can lead to a reduction in inflammation markers and dysbiosis, ultimately enhancing the quality of life for individuals with ulcerative colitis. This type of dietary approach supports a healthier gut environment, which is essential for managing symptoms associated with this condition. By prioritizing fiber-rich foods while minimizing fat intake, patients can promote beneficial changes in their gut microbiome. These changes help to restore balance among gut bacteria, which can alleviate some of the inflammation typically seen in ulcerative colitis. As a result, individuals may experience fewer flare-ups and improved overall well-being, highlighting the importance of dietary choices in managing chronic inflammatory conditions (73).

Dietary fibers play a crucial role in lowering the risk of inflammatory bowel disease (IBD). Research has revealed a significant inverse relationship between dietary fiber intake and the incidence of Crohn’s disease. Studies indicate a robust protective association specifically linked to the consumption of fruit-derived dietary fibers, which appear to be beneficial for both Crohn’s disease and ulcerative colitis. These findings suggest that incorporating fiber-rich foods, particularly fruits, into the diet may help mitigate the risk of developing these conditions. The beneficial effects of dietary fiber likely stem from its ability to promote a healthy gut microbiome and reduce inflammation, both of which are vital in managing and preventing IBD. As awareness of these associations grows, increasing fiber intake can be seen as a proactive measure for individuals at risk of or currently managing inflammatory bowel diseases (74).

A diet high in fiber may help decrease inflammation by altering gut permeability and pH levels. This adjustment within the gut can lead to reduced inflammatory responses, which might also positively influence neurotransmitter levels in the brain. Consequently, these changes can help alleviate symptoms associated with depression. This relationship underscores the idea that increasing dietary fiber not only helps combat inflammation but may also support better mental health outcomes. By fostering a balanced gut microbiome and lowering inflammation, dietary fiber could play a role in enhancing mood and emotional stability. Therefore, it’s clear that incorporating fiber-rich foods into one’s diet can have far-reaching benefits, contributing both to physical health by reducing inflammation and to mental well-being by potentially mitigating depressive symptoms (75). The key points of this section are categorized in Table 5.

**Table 5:** Key points of fiber-rich diet influence on inflammatory disease.

Topic	Key points
Chronic Inflammatory Diseases	- Chronic inflammation linked to CVD, type II diabetes, and metabolic syndrome, leading to reduced life quality.
Managing Inflammation	- Anti-inflammatory diet (fruits, vegetables, whole grains), exercise, stress management, and sleep are key strategies.
Fiber’s Role in Inflammation	- Fiber-rich diet (especially from fruits) alters gut microbiome to lower inflammation and reduce risk of CVD & IBD.
Gut Health & IBD	- High fiber diet reduces inflammation markers and improves gut health, beneficial for ulcerative colitis and Crohn’s disease.
Mental Health	- Fiber may reduce gut inflammation, improving brain function and alleviating depression symptoms.
Fiber’s Protective Benefits	- Fruit-derived fibers help manage chronic inflammation and are protective against diseases like IBD and depression.

## 2 Challenges and limitations

The increasing body of evidence linking dietary fiber to improved health outcomes in chronic conditions such as CVD, type II diabetes, colon cancer, and inflammation has sparked widespread interest in fiber-based interventions. However, despite its promise, the research in this area faces several challenges, including limitations in study design, gaps in mechanistic understanding, and the need for further exploration of fiber’s long-term impacts. These limitations point to key areas where additional research is needed to fully grasp the extent of fiber’s benefits and to refine its application in clinical practice.

One of the major challenges lies in the variability of fiber types and their distinct physiological effects. Dietary fiber is commonly classified into soluble and insoluble types, yet many studies do not distinguish between these different categories, which may play distinct roles in disease prevention and management. Soluble fibers, found in foods such as oats, legumes, and fruits, are generally thought to improve cholesterol levels, regulate blood sugar, and enhance cardiovascular health. On the other hand, insoluble fibers, typically found in vegetables, seeds, and whole grains, are known for their role in promoting digestive health and preventing constipation. The broad categorization of fiber in many studies limits the ability to pinpoint which specific type of fiber is most beneficial for a given condition. Given that different types of fiber may interact differently with gut bacteria and metabolic pathways, future research should focus on isolating the distinct effects of soluble and insoluble fibers to better inform dietary recommendations. This differentiation will enable healthcare providers to tailor fiber-based interventions to specific health concerns, optimizing patient outcomes (22).

Another critical limitation is the lack of long-term studies exploring fiber’s sustained effects on chronic disease progression. Much of the existing research focuses on short-term outcomes such as improvements in blood glucose control or lipid profiles, but these effects do not always translate into long-term disease prevention. While many studies show promising results for fiber’s role in controlling risk factors for type II diabetes, CVD, and other chronic conditions, there is a need for randomized controlled trials (RCTs) that track the long-term benefits of fiber intake over several years. This is especially important for diseases such as colon cancer, where diet and lifestyle factors play a pivotal role in disease development over time. To develop more comprehensive treatment plans, it is essential to understand how fiber’s protective effects evolve over the lifespan, and whether continued intake is required to sustain these benefits (76). The mechanisms by which fiber exerts its beneficial effects on health remain poorly understood. While there is consensus that fiber improves metabolic health by regulating blood sugar, reducing cholesterol levels, and lowering systemic inflammation, the biological pathways driving these changes are still being explored. One area of active investigation is the role of the gut microbiome in mediating fiber’s effects. Fiber serves as a substrate for fermentation by gut bacteria, producing SCFAs such as butyrate, acetate, and propionate, which have been linked to improved insulin sensitivity and reduced inflammation. However, the exact mechanisms through which SCFAs influence metabolic and immune responses are not yet fully defined. Additionally, while fiber’s impact on gut microbiota is well-documented, it is unclear how much of its beneficial effects on diseases like Type II diabetes and CVD are directly due to changes in gut microbiota composition versus other factors like improved digestive function or reduced systemic inflammation. Addressing these questions in future research will be critical to understanding fiber’s full therapeutic potential (77).

Colon cancer prevention is another area in which fiber’s role remains debated. Numerous observational studies suggest that high-fiber diets may reduce the risk of colon cancer, but the evidence from clinical trials is less conclusive. The protective effects of fiber are thought to be mediated by its ability to produce SCFAs during fermentation, which help lower colon pH and may inhibit carcinogenic pathways. However, not all studies have found significant reductions in cancer incidence associated with fiber intake, and some have suggested that other dietary factors, such as fat or protein intake, may play a more substantial role. To strengthen the evidence base, large-scale, long-term studies specifically examining fiber’s direct role in colon cancer prevention are needed (64).

In the clinical context, it is important to evaluate the effectiveness of fiber in comparison to alternative interventions for managing chronic conditions. For instance, fiber intake is well-established for its role in improving blood glucose control and insulin sensitivity in individuals with Type II diabetes. However, alternative dietary approaches, such as low-carbohydrate or ketogenic diets, have also shown significant effects on blood sugar regulation. These diets work by reducing the intake of carbohydrates, which helps lower blood glucose levels and can improve insulin resistance. While fiber’s benefits are largely attributed to its ability to slow digestion and enhance insulin sensitivity, low-carbohydrate and ketogenic diets achieve similar outcomes through different mechanisms. Given the growing interest in these alternative diets, comparing their long-term effectiveness, sustainability, and overall health benefits against fiber-rich interventions is crucial for providing optimal clinical recommendations. Studies that directly compare these strategies in clinical trials could help clarify the most effective approach for managing Type II diabetes, as well as other chronic conditions such as cardiovascular disease and obesity. These diets are often more restrictive than high-fiber diets and may provide quicker results for some individuals, but they come with their own set of challenges, such as increased risk of nutrient deficiencies or difficulties with long-term adherence. In clinical practice, it may be necessary to combine fiber with other interventions, such as physical activity, pharmacological treatments, or other dietary adjustments, to achieve optimal outcomes in managing chronic conditions. A head-to-head comparison of fiber versus these alternative interventions could provide valuable insights into the most effective treatment strategies for patients, especially those with comorbidities like obesity, hypertension, and hyperlipidemia. Additionally, while pharmacological treatments like statins are commonly prescribed for individuals with elevated cholesterol and cardiovascular risk, it remains unclear whether fiber could serve as a more sustainable and cost-effective option for long-term management. Some studies suggest that dietary fiber, particularly soluble fiber, may provide comparable cholesterol-lowering effects to statins, with fewer side effects and lower costs. This raises the question of whether fiber-based interventions should be considered as an adjunct or even an alternative to pharmacological treatments in certain populations (78).

Finally, a broader consideration in fiber research is its integration into dietary guidelines and public health recommendations. Despite its potential, dietary fiber intake remains suboptimal in many populations. Public health campaigns and clinical practice guidelines need to focus not only on the benefits of fiber but also on strategies to overcome common barriers to fiber consumption, such as limited access to whole foods, lack of education on fiber-rich diets, and food preferences that may be shaped by culture or socioeconomic factors. Further research into effective public health interventions that increase fiber intake across diverse populations will be essential to translate these scientific findings into meaningful changes in health outcomes (79).

In summary, while dietary fiber has a well-documented positive impact on a range of chronic diseases, significant challenges remain in understanding its full potential. There are still substantial knowledge gaps regarding the specific mechanisms through which fiber impacts health, the long-term effects of fiber intake, and its comparative effectiveness relative to other interventions. Filling these gaps will require more targeted research, particularly longitudinal studies and clinical trials that distinguish between different types of fiber and their distinct effects on chronic disease. By addressing these limitations, we can better integrate fiber into clinical practice and public health strategies to improve outcomes for patients with cardiovascular disease, Type II diabetes, colon cancer, and other inflammatory conditions.

## 3 Conclusion

Dietary fiber, encompassing both soluble and insoluble forms, plays a pivotal role in mitigating the risk of several chronic diseases, including cardiovascular disease (CVD), type II diabetes, obesity, and inflammation. Its benefits extend beyond digestive health, supporting weight management, enhancing insulin sensitivity, and regulating nutrient absorption. Increasing fiber intake through foods like fruits, vegetables, whole grains, legumes, and nuts can contribute to better overall health, including improved blood sugar regulation and enhanced gut function. Despite these well-established benefits, significant gaps remain in our understanding of the precise mechanisms through which different types of fiber influence health, as well as the ideal amounts needed to prevent or manage disease. Factors such as variations in individual gut microbiota and lifestyle habits complicate our ability to offer clear, one-size-fits-all recommendations.

To bridge these gaps, future research should include diverse populations and conduct longer-term studies to compare the effects of fiber with other dietary approaches, such as low-carb or Mediterranean diets. Delving deeper into the interactions between fiber, the gut microbiome, and metabolic health could uncover new therapeutic avenues for chronic disease management. As the body of evidence expands, dietary guidelines can become more refined, allowing for the development of more personalized recommendations that cater to individual health profiles and conditions.

To capitalize on the health benefits of fiber, public health initiatives must prioritize education on the importance of increasing fiber intake. This includes making it easier for individuals to incorporate fiber-rich foods into their diets, with a focus on practical strategies that promote whole-food sources such as vegetables, fruits, whole grains, and legumes. Healthcare providers should also play a key role in embedding fiber recommendations into chronic disease management, tailoring advice to each patient’s unique health needs. Additionally, policy changes are necessary to increase access to affordable, nutritious, fiber-rich foods, particularly in underserved areas, where food insecurity often limits dietary options. By improving both knowledge and access, we can empower individuals to take charge of their health, reduce disease risk, and improve overall public well-being.
